# Surveillance of Barrett's Esophagus Patients in an Expert Center is Associated With Low Disease‐Specific Mortality

**DOI:** 10.1002/ueg2.12759

**Published:** 2025-02-13

**Authors:** Judith Honing, W. Keith Tan, Victor Yan Zhe Lu, Vlasios Gourgiotis, Isaac M. Gianfrancesco, Alina A. Schumacher, Shriya Vishwanathan, Calvin Cheah, Ines Modolell, Vijay Sujendran, Rebecca C. Fitzgerald, Massimiliano di Pietro

**Affiliations:** ^1^ Early Cancer Institute University of Cambridge Cambridge UK; ^2^ Erasmus Medical Center Erasmus University Rotterdam The Netherlands; ^3^ Department of Gastroenterology Cambridge University Hospital NHS Foundation Trust Cambridge UK; ^4^ School of Clinical Medicine University of Cambridge Cambridge UK; ^5^ Cambridge Oesophagogastric Centre Cambridge University Hospital NHS Foundation Trust Cambridge UK

**Keywords:** Barrett's esophagus, esophageal adenocarcinoma, high‐grade dysplasia, intestinal metaplasia

## Abstract

**Introduction:**

Specialist guidelines recommend endoscopic surveillance for Barrett's esophagus to reduce mortality related to esophageal adenocarcinoma, but the setting for optimal Barrett's esophagus monitoring is unclear. We assessed progression rate and disease‐specific mortality in a large cohort of patients followed up at a single Barrett's esophagus expert center.

**Methods:**

For this prospective longitudinal single center cohort study, we recruited patients with a previous diagnosis of Barrett's esophagus between 2004 and 2022. Endoscopists were trained in Barrett's esophagus surveillance standards and image‐enhanced techniques, and biopsies were reviewed by expert pathologists. Exclusion criteria were a single surveillance endoscopy, high‐grade dysplasia, or esophageal adenocarcinoma at or within 12 months from index endoscopy and patients with < 12 months follow‐up. The primary outcome was the neoplastic progression rate of Barrett's esophagus with intestinal metaplasia to high‐grade dysplasia/esophageal adenocarcinoma. Secondary outcomes included cancer stage and disease‐specific mortality, risk factors for progression and progression rate in patients with Barrett's esophagus with only gastric metaplasia or irregular z‐line and intestinal metaplasia (IZL‐IM).

**Results:**

A total of 1932 patients were recruited, of which 969 were included in the primary analysis with a median follow‐up of 5.8 years. Of these, 109 developed high‐grade dysplasia or esophageal adenocarcinoma with a progression rate of 1.63%/year. Overall, 48 patients received an esophageal adenocarcinoma diagnosis, of which 89,5% (43/48) had stage 1%, and 0.3% patients (3/969) had disease‐specific mortality. Multivariate analysis showed that age, alcohol consumption, esophagitis, Barrett's esophagus length, hiatus hernia length, low‐grade dysplasia and neutrophil/lymphocyte ratio were risk factors for progression. The rate of progression in patients with Barrett's esophagus—gastric metaplasia or IZL‐IM was 0.06%/year.

**Conclusions:**

Endoscopic surveillance in an expert Barrett's esophagus center leads to a high neoplastic progression rate, and a low rate of disease‐specific mortality. Further research to correlate disease‐specific mortality and cancer stage with dysplasia detection rate is warranted to develop diagnostic quality indicators specific for Barrett's esophagus.

## Graphical abstract

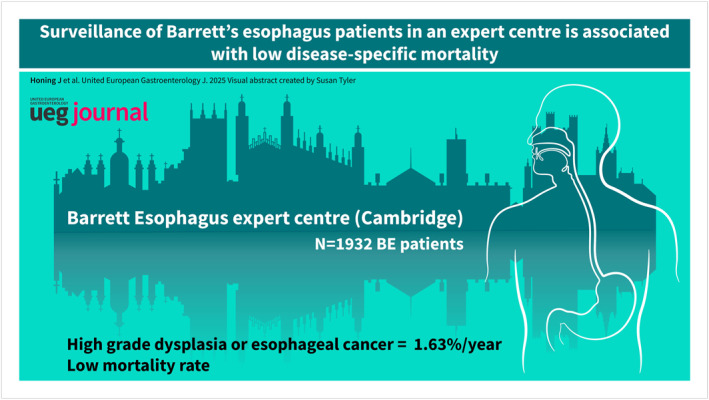

1

**Summary**

- Established knowledge
  ◦ Barrett's esophagus (BE) has a low rate of neoplastic progression, varying between 0.78% and 1.20% in prospective studies in community and tertiary centers.
  ◦ Surveillance in expert centers associates to high rate of adherence to the Seattle protocol, but data on the impact on mortality by esophageal adenocarcinoma (EAC) is scarce.
- Significant new findings
  ◦ In this prospective longitudinal study in a single BE expert center, the neoplastic progression rate to high‐grade dysplasia or EAC was 1.63%.
  ◦ Disease‐specific mortality was 0.3% in the overall cohort and 6.3% among cases diagnosed with EAC within the study period.
  ◦ Further studies on the correlation between disease‐specific mortality and setting of surveillance (expert vs. non‐expert centers) are needed to identify quality indicators for BE surveillance.

## 2 Introduction

The incidence of esophageal adenocarcinoma (EAC) has risen dramatically over the last 40 years [1]. Up to 40% of patients present with metastatic disease at diagnosis, resulting in a poor overall 5‐year survival of only 20% [2]. Barrett's esophagus (BE), a metaplastic condition of the esophageal mucosa, which occurs in response to chronic gastroesophageal reflux, is the only known premalignant precursor to EAC [3]. BE is believed to progress to EAC through intermediate dysplastic stages, that is low‐grade dysplasia (LGD) and high‐grade dysplasia (HGD), which represent the basis for endoscopic surveillance and endoscopic eradication therapy (EET) of pre‐invasive and early invasive neoplastic disease [4]. However, the estimated annual progression rate of BE to cancer is only 0.33%/year [5] and approximately 95% of EAC patients do not have a previous BE diagnosis [6, 7].

Although endoscopic surveillance is recommended by all specialty guidelines [8, 9, 10], there is no randomised evidence that surveillance improves patient outcomes. Two population‐based case‐control studies from the Netherlands and Northern Ireland showed that the hazard ratio for death by EAC in patients with BE undergoing endoscopic monitoring was 0.79 (95% CI 0.64–0.92) and 0.65 (95% CI 0.45–0.95), respectively [11, 12]. However, two case‐control studies on the US Veteran community showed that previous endoscopic surveillance had limited or no protective effect on disease‐specific mortality in people diagnosed with EAC [13, 14].

The conflicting data on the benefit of surveillance might derive from the varying degree of adherence to recommended protocols, different expertise of endoscopists performing diagnostic procedures and inconsistency in the inclusion criteria into studies. Verbeek et al. showed that only when BE monitoring is adequate with adherence to optimal surveillance intervals it reduces mortality from EAC [15]. Another reason for the lack of benefit from endoscopic surveillance is low adherence to the Seattle protocol. A US retrospective study showed that less than 50% of endoscopists perform adequate mapping biopsies in standard clinical practice, with rates dropping to just 10% for patients with BE of 9 cm or longer [16]. In contrast, when surveillance is performed in expert centers or by endoscopists as part of a dedicated BE surveillance service, adherence to Seattle protocol biopsies is high (78%–85% compared to 19%–66% in non‐expert centers) [17, 18]. Finally, population‐based registries of histopathology records have the caveat of including patients with ultra‐short BE or even without pathological evidence of intestinal metaplasia (IM) [11, 12].

To address these issues, we conducted a longitudinal prospective cohort study in a single expert center. The primary objective was to calculate the progression rate to HGD or in BE patients managed in an expert center. The secondary objectives included disease‐specific mortality in expert center, risk factors for progression, the progression rate to HGD/EAC in patients with BE without IM and irregular Z‐line (IZL) with IM.

## 3 Methods

### 3.1 Study Design

This prospective single center study was conducted over a period of 24 years from 1997 to 2022 and according to STROBE guidelines [19]. The Cambridge Barrett's Registry study was approved by the local ethics committee of Cambridge University Hospitals (LREC01/197). Eligible patients were invited to join the study, and any patients newly diagnosed during the study period were asked for consent to join the study prospectively. Endoscopy and pathology data were prospectively entered on a dedicated database (initially Microsoft Access and later customised web‐based platform). Missing data at the time of the analysis were retrieved from Addebrookes hospital's electronic patient medical record system. Missed EAC diagnoses in patients lost in follow‐up or who declined further surveillance were identified via interrogation of International Classification of Diseases (ICD) codes and upper GI multidisciplinary team (MDT) records.

### 3.2 Inclusion and Exclusion Criteria

Because of the change in disease definition over time, patients were eligible for the study if they had either evidence of columnar‐lined esophagus (CLE) extending proximally from the gastroesophageal junction or IM on esophageal biopsies. However, for the primary outcome, BE was defined by current guidelines, that is, endoscopic extent of CLE at least 1 cm and the presence of IM on biopsies. Excluded from this analysis were patients with a single diagnostic endoscopy, evidence of HGD or EAC within 12 months from index endoscopy, esophageal squamous cell carcinoma (ESCC) and previous treatment for any type of dysplasia at the time of referral. We analyzed separately rates of neoplastic progression in patients with IZL (CLE < 1 cm) with IM (IZL‐IM) and BE with gastric metaplasia only (BE‐GM).

### 3.3 Prospective Data Collection

An endoscopic surveillance program dedicated to the surveillance of BE was performed for all patients according to national guidelines [20]. Endoscopy procedures and biopsy sampling were performed by experienced research endoscopists who were trained in image‐enhanced endoscopy using high‐definition white light endoscopy (HD‐WLE) and chromoendoscopy. Biopsies were taken according to the Seattle Protocol [21]. All biopsies were reviewed by expert pathologists and graded according to the Vienna classification [22]. All cases of dysplasia were reviewed by an expert gastrointestinal pathologist. All data were uploaded onto a customised online platform.

Demographic and endoscopic data were collected prospectively. Additional data regarding medication and exposure were prospectively collected using questionnaires or retrospectively from the electronic patient file where missing. Age was calculated at the time of index endoscopy. For progressors, the maximum BE length was calculated as the median of the maximum length measured at each endoscopy until HGD/EAC was diagnosed. For non‐progressors, the median value of the maximum length until the last follow‐up was used. Cancer diagnosis and disease‐specific mortality was ascertained via electronic medical records, upper GI multidisciplinary meeting and regional cancer registration (JCIS). Full blood count (FBC) data were retrieved from the patients’ file to determine the neutrophil to lymphocyte ratio (NLR). To reduce bias, FBC records were selected that were unrelated to hospital admission (e.g. due to an infection), and FBC records were as close as possible to baseline OGD. If the latter was not possible, FBC records were selected that were no later than 12 months from the last EGD before progression. Patients with known hematological diseases were excluded.

### 3.4 Statistical Analysis and Ethical Approval

The primary outcome was the progression to HGD/EAC in patients with BE with IM and at least 1 cm in length. Secondary outcomes were disease‐specific mortality (DMS), time to progression to HGD/cancer in patients with different disease extents (BE‐GM/IZL‐IM, BE 1–2 cm with IM, BE 3–9 cm with IM and BE ≥ 10 cm with IM), and factors predictive of neoplastic progression. Total surveillance time was reported as the time from index endoscopy to either, last surveillance endoscopy, date of progression to HGD/cancer, or until EET treatment in a small subset of patients who developed LGD.

Normality testing was performed using the Shapiro‐Wilk test. Continuous variables were represented as means with standard deviation (SD) or medians and interquartile range (IQR) where appropriate. A Cox proportional hazard model was used to identify predictors of neoplastic progression and covariates with a *p*‐value of *p* < 0.1 were included in a backward elimination model. Values were presented as hazards ratio (HR) with 95% confidence interval (CI). Kaplan–Meier estimates were used to compare the probability of progression to HGD/EAC among different BE groups and Log‐rank *p*‐value was used to assess for statistical significance. A *p*‐value of < 0.05 denoted statistical significance. Annual progression rate was calculated by dividing the number of events (progression) by the total person‐time of follow‐up. DSM was calculated as the number of events (EAC‐related death) divided by the number of cases at risk; this was an intention‐to‐treat analysis, meaning that we accounted for all patients who had surveillance, including those who declined further surveillance at any interval after the second endoscopy. Statistical analysis was performed using IBM SPSS Statistics version 29.0 and R version 4.2 (Vienna, Austria).

## 4 Results

**FIGURE 1:**
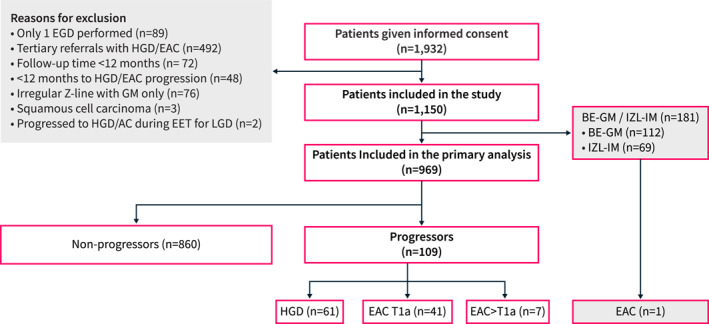
Patient flowchart. Of the 1932 patients who consented to the Cambridge Barrett's registry, 782 did not meet the inclusion criteria for this study. The 181 Barrett's esophagus with gastric metaplasia only (BE‐GM) and irregular Z‐line with IM (IZL‐IM) were only included in the secondary analysis. 109 patients with BE (11.2%) progressed during the study period to HGD/EAC. A single patient with minimal Barrett's disease progressed to cancer. EAC, esophageal adenocarcinoma; EGD, Esophago‐gastroduodenoscopy; EET, endoscopic eradication therapy; GM, gastric metaplasia; IMC, intramucosal cancer.

Between 1997 and 2022, 1932 patients with BE were identified by searching the Cambridge Barrett's registry database or were diagnosed with BE during the study period (Figure 1). Patients were excluded from the primary outcome analysis if they had only one EGD performed (*n* = 89), HGD or EAC < 12 months from index endoscopy (*n* = 492), IZL with no intestinal metaplasia (*n* = 76), < 12 months follow‐up time (*n* = 72), < 12 months before progression to HGD or EAC (*n* = 48), esophageal squamous cell carcinoma (*n* = 3), started treatment for LGD then progressed to HGD or EAC during treatment (*n* = 2). The remaining 1150 eligible patients had their medical records were reviewed; we identified 181 patients with BE‐GM or IZL‐IM, who were excluded from the primary endpoint analysis. After this step, 969 patients were included for the analysis, of which 860 were non‐progressors and 109 progressed to HGD or EAC.

Baseline demographics for progressors and non‐progressors are presented in Table 1. The median follow‐up time was 5.8 years (IQR 3–9.6), with a total follow‐up time of 6706 years, which equals to an annual neoplastic progression rate of 1.63 per‐person year. Compared to non‐progressors, the progressors had a longer maximum BE length (median 5 vs. 3) and were more likely to have LGD at baseline endoscopy [22.9% (25/109) versus 9.1% (78/860)]. All progressors were taking a PPI, with the majority using omeprazole [51.3% (56/109)]. Detailed characteristics of the diagnosis and treatment of the progressors are shown in Table 2.

**TABLE 1:** Demographical and clinical characteristics.

	Progressors (*n* = 109)	Non‐progressors (*n* = 860)
Age (years)		
mean ± SD	63.0 ± 10.1	62.3 ± 15.9
At Progression	68.8 ± 9.35	
Gender		
Male	86 (78.9%)	577 (67.1%)
Female	23 (22.1%)	282 (32.8%)
Missing		1 (0.10%)
BMI		
Mean ± SD	28.7 ± 6.1	28.2 ± 5.8
Alcohol use		
Yes	67 (61.5%)	250 (29.1%)
No	14 (12.8%)	283 (33.0%)
Unknown	28 (25.6%)	327 (37.9%)
Smoking status		
Never smoker	31 (28.4%)	275 (31.9%)
Ex‐smoker	47 (43.1%)	205 (23.8%)
Current smoker	9 (8.3%)	103 (12.0%)
Unknown	22 (20.2%)	277 (32.2%)
Hiatus hernia (cm)		
Median ± IQR	4 (3–6)	3 (IQR 0–5)
Surveillance time (years)		
median ± IQR	5.03 (2.4–8.0)	5.9 (3.0–9.9)
Number of endoscopies		
median (IQR)	4 (2–7)	4 (3–7)
Maximum length of Barrett's esophagus (cm)		
median ± IQR	5 (4–8)	3 (2–6)
LGD at baseline endoscopy	25 (22.9%)	78 (9.1%)
Esophagitis signs		
Yes	67 (61.5%)	299 (34.7%)
No	42 (38.5%)	404 (47.0%)
Unknown	0 (0%)	157 (18.2%)
Taking PPI		
Esomeprazole	28 (25.6%)	136 (15.8%)
Omeprazole	56 (51.3%)	294 (34.1%)
Lansoprazole	15 (13.8%)	205 (23.8%)
Pantoprazole	7 (6.4%)	29 (3.4%)
Rabeprazole	1 (0.9%)	4 (0.5%)
None	0	28 (3.2%)
Unknown	2 (1.8%)	165 (19.1%)
Aspirin		
Yes	26 (23.8%)	303 (35.2%)
No	72 (66.1%)	375 (43.6%)
Unknown	11 (10.1%)	182 (21.1%)
NSAIDs		
Yes	23 (21.1%)	324 (37.7%)
No	75 (68.8%)	364 (42.3%)
Unknown	11 (10.1%)	172 (20.0%)
Statins		
Yes	42 (38.5%)	333 (38.7%)
No	56 (51.4%)	344 (39.9%)
Unknown	11 (10.1%)	183 (21.2%)
NLR		
Median (IQR)	2.6 (1.7–3.5)	2.1 (1.6–2.8)

Abbreviations: BMI, body‐mass index; IQR, interquartile range; LGD, low‐grade dysplasia; NLR, neutrophil to lymphocyte ratio; NSIADs, non‐steroidal anti‐inflammatory drugs; PPI, proton‐pump inhibitor; SD, standard deviation.

**TABLE 2:** Characteristic progressors (*N* = 109).

Baseline pathology	
NDBE	71 (65.1%)
IND	12 (11%)
LGD	26 (23.9%)
Time from prior endoscopy to progression (months)^a^	
For all progressors	14.4 (6.5–27.8)
NDBE at index	24.8 (7.2–33.9)
IND at index	19.7 (7.8–26.1)
LGD at index	7.7 (3.3–13.6)
Number of endoscopies to progression^a^	
NDBE	4 (2–7)
IND	4 (2–8)
LGD	4 (2–8)
Targeted or random biopsy at progression	
Random	52 (47.7%)
Targeted	56 (51.4%)
Missing	2 (0.9%)
Active esophagitis	
NDBE at index	44 (62.0%)
IND at index	8 (66.7%)
LGD at index	15 (55.7%)
Highest pathology	
HGD	61 (56.0%)
Intramucosal adenocarcinoma	41 (37.6%)
Adenocarcinoma (≥ pT1b)	7 (6.4%)
Treatment	
Endoscopic treatment	95 (85.6%)
Surgery	7 (6.3%)
None/surveillance	4 (3.7%)
Palliative	1
Missing	2

Abbreviations: HGD, high‐grade dysplasia; IND, indefinite for dysplasia; LGD, low‐grade dysplasia; NDBE, non‐dysplastic Barrett's esophagus.

^a^ Median ± IQR.

The vast majority of progressors in our cohort were accounted for by early neoplasia [HGD in 56.0% (61/109) and intramucosal carcinoma in 38.5% (41/109) of progressors]. Most of them (85.6%, 95/109) were treated endoscopically using EMR, ESD, RFA or a combination. The median time between last surveillance endoscopy and any progression was 14.4 months (6.5–27.8), while the median time to progression to cancer stage > 1 was 18.0 months (4.2–33.8). In total, 48 had EAC, of which 89.5% (43/48) had stage I, 2% (1/48) had stage II, 6% (3/48) had stage III, 2% (1/48) had stage IV. Among patients with a diagnosis of cancer, only 6.3% (3/48) had disease‐specific mortality. One patient was diagnosed with stage III cancer during surveillance in 2005 but had limited therapeutic options due to severe comorbidities upon cancer diagnosis. The other two were diagnosed with stage III and IV EAC at the early stages of the study in 2001 and 2002, respectively. We used a Cox‐proportional hazard model to identify predictors of neoplastic progression (Table 3). Multivariable analysis showed that age (HR 1.04 CI 1.01–1.07), alcohol (HR 2.90 CI 1.41–5.93); esophagitis (HR 6.51 CI 3.31–12.82); hiatus hernia length (1.08 CI 1.02–1.15), maximum Barrett's length (HR 1.12 CI 1.03–1.23), LGD at baseline (HR 2.16 CI 1.09–4.26), and NLR (HR 1.51 1.26–1.80) were significant risk factors for progression, whereas NSAIDs use exerted a protective effect (HR 0.29 CI 0.15–0.58).

**TABLE 3:** Univariate and Multivariate logistic regression analysis for factors associated with progression.

*N* = 969	*Univariate*			*Multivariate*		
	Hazard ratio	95% CI	*p*‐value	Hazard ratio	95% CI	*p*‐value
Gender^a^	1.63	1.03–2.59	0.034			
Age	1.01	1.00–1.01	0.022	1.04	1.01–1.07	0.008
Smoking						
Never	1					
Ex smoker	1.88	1.20–2.97				
Smoker	0.70	0.33–1.47	0.0027			
Alcohol	4.02	2.26–7.17	< 0.001	2.90	1.41–5.93	0.004
BMI	1.01	0.98–1.05	0.459			
Esophagitis	1.74	1.19–2.56	0.005	6.51	3.31–12.82	< 0.001
Aspirin	0.47	0.30–0.73	< 0.001			
NSAID	0.34	0.21–0.54	< 0.001	0.29	0.15–0.58	< 0.001
Statin	0.88	0.59–1.31	0.513			
Hiatus hernia	1.05	1.01–1.09	0.007	1.08	1.02–1.15	0.005
Maximum Barrett's (cm)	1.15	1.09–1.21	< 0.001	1.12	1.03–1.23	0.012
LGD at baseline	2.28	1.46–3.58	< 0.001	2.16	1.09–4.26	0.027
NLR	1.56	1.38–1.78	< 0.001	1.51	1.26–1.80	< 0.001

Abbreviations: BMI, body mass index; NSAID, non‐steroidal anti‐inflammatory drugs; PPI, proton pump inhibitor.

^a^ Data shown are for the association between males and progression.

**FIGURE 2:**
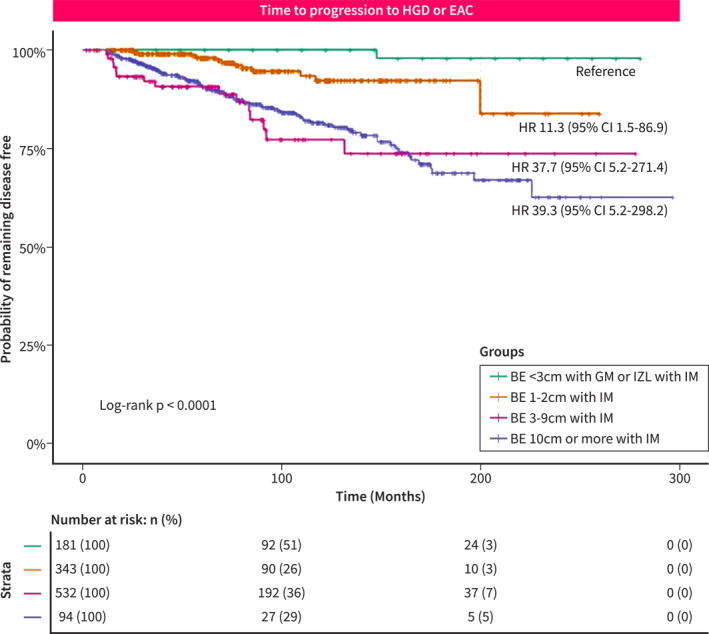
Kaplan–Meier estimates of the probability of progression to HGD or EAC among the different BE subgroups. Vertical marks indicate the time of censoring. The estimate of the hazard ratio was based on a Cox model stratified according to the length of BE and presence of intestinal metaplasia (IM) and adjusted for age and gender. There was an indication of non‐proportional hazards for the longer segment BE groups (BE 3–9 cm and BE ≥ 10 cm). BE‐GM or irregular Z‐line (IZL) with IM was used as a reference.

For the secondary analysis, we looked at patients with BE‐GM or IZL‐IM (*n* = 181) who were monitored for a total of 1701 person‐years of follow‐up. Among those, none progressed to HGD, but 1 patient developed EAC and subsequently died of this disease, leading to a neoplastic progression rate of 0.06% per‐person year and a disease‐specific mortality of 0.5%. We then looked at the combined effect of length and presence of IM on progression and stratified patients into 4 groups, that is, BE‐GM/IZL‐IM (*n* = 181), BE 1–2 cm with IM (*n* = 343), BE 3–9 cm with IM (*n* = 532) and BE ≥ 10 cm with IM (*n* = 94). With the probability of remaining free of HGD or EAC in the minimal BE group as the reference, we found an increasing probability of progression in the BE > 1 cm with IM (Figure 2). Interestingly, when comparing patients with long segment BE 3–9 cm with IM versus ultralong BE ≥ 10 cm, the overall rate of progression was similar.

## 5 Discussion

This prospective longitudinal study demonstrates that endoscopic surveillance of patients with BE performed at an expert center is associated with a low rate of advanced cancer and disease‐specific mortality.

A meta‐analysis published over 10 years ago showed that the estimated annual progression rate of BE to EAC is 0.33% (95% CI: 0.28%–0.38%) per year; this meta‐analysis was mostly based on retrospective studies [5]. Recent prospective studies showed an annual rate of progression to HGD/EAC varying between 0.78% and 1.2% (Supplementary Table S1) [23, 24, 25]. Our study showed a higher annual rate of progression of 1.63% per year, despite a similar rate of index LGD. While this could depend on geographical differences between studies, the difference might be explained by the relative older population in our cohort aged 62–63 years at index compared to 55–57 years in the studies with progression rates around 0.80% [23, 24]. Another explanation might be the high detection rate of neoplasia in the expert setting of our study, with strict adherence to the Seattle protocol and the systematic use of image‐enhanced endoscopy. Among the 3 previous prospective studies, with similar sample sizes, one was tertiary‐center based, one was community‐based and one was mixed tertiary and community‐based. Recent data from the UK and the Netherlands show that surveillance performed in a dedicated BE service is associated with higher adherence to the Seattle protocol compared to standard endoscopy service, although the dysplasia detection rate was not uniformly increased in a dedicated BE service [26, 27].

Despite high rates of HGD or EAC detection, the disease‐specific mortality in our cohort was only 0.3% overall, and 6.3% among all EAC cases diagnosed within the study period. It is important to note that to calculate disease‐specific mortality, we conducted an intention‐to‐treat analysis, which included patients who declined surveillance and those who were unfit for treatment. In a prospective study with similar size and mixed expert/non‐expert setting, Kastelein et al. reported that 17% of EAC cases diagnosed on surveillance had disease‐specific mortality [25]. Two retrospective UK studies looking at the impact of surveillance with Seattle protocol biopsies showed that the rate of disease‐specific mortality in patients diagnosed with EAC during surveillance was 30%–50% [28, 29]. These rates of cancer related mortality among BE patients on surveillance suggest that surveillance outside dedicated services does not reduce mortality from EAC in a significant proportion of cases diagnosed with cancer. Our data linking high detection rates of HGD or EAC diagnosis with improved cancer outcomes have important implications for the development of quality indicators in BE surveillance. However, it is difficult to extrapolate a diagnostic key performance indicator from our data and future multicenter studies should further investigate the correlation between annual HGD or EAC diagnosis rate and disease‐specific mortality.

Our data on the risk factors for progression is mostly confirmatory of previous research, in that we have found that BE length, previous diagnosis of esophagitis and hiatus hernia correlated with the risk of progression. With regard to lifestyle and pharmacological exposure, we found that alcohol consumption increased the risk of progression and NSAIDs exerted a protective effect, while smoking did not correlate with the risk of progression. There is inconsistency among studies on the impact of alcohol and smoking on the cancer risk. A meta‐analysis from 20 studies found that individuals who had ever smoked had an OR of 1.47 (95% CI, 1.09–1.98) for progression, while alcohol consumption did not correlate to the risk of progression [30]. A recent prospective study, however, is in agreement with our data that smoking did not increase the risk [23]. It must be noted that in our study life‐style and pharmacological exposures were not fully recorded prospectively, and required manual retrieval of historical records and had 10%–37% of missing data; therefore, we cannot exclude this has impacted the strength of the correlations found. Furthermore, our study also provides external validation for high NLR as a clinical biomarker for cancer risk. A previous small retrospective study including 324 BE patients of which 13 progressed showed that high NLR was associated with 3‐fold increase in the risk of progression with an optimal cut‐off of 2.4 [31]. In our dataset, NLR was independently associated with a higher hazard of progression to HGD or EAC, but the optimal cut‐off for risk stratification needs to be further explored.

Finally, our study provides further insight into the risk of progression in patients who do not fully meet either length or histologic criteria for BE diagnosis (BE‐GM and IZL‐IM). Among 181 individuals within this group, we observed only one case of EAC in a patient who had IZL‐IM, giving an overall annual progression rate of 0.06%/year. These data agree with previous evidence from the Northern Irish population study showing that the risk of progression in individuals with BE‐GM is 5‐fold lower than patients with IM, with an annual HGD or EAC incidence of 0.07% [11]. More recently, Black et al. provided evidence than GM has significantly lower mutational burden compared to IM and lacks genomic hallmarks of EAC [32]; a proportion of the patients investigated in this study was included in the BE with GM cohort presented here. Overall, our data are in support of current policy of not recommending surveillance in cases with IZL‐IM and those without histologically proven IM within BE segment < 3 cm on two consecutive endoscopies. Furthermore, our data demonstrated that, while the length of BE is a risk factor for neoplastic progression, patients with ultralong BE (10 cm or more) did not have a higher progression rate than those with long segment but < 10 cm, suggesting that similar surveillance intervals in these two groups are appropriate [8].

This study has several limitations. First, we did not have a control group of non‐dedicated BE service to compare outcome data; however, historical data represent a valid benchmark to gauge the performance of our expert endoscopy service. Second, despite the prospective nature of our study, there was a degree of missing data on lifestyle and pharmacological exposures, which was compensated by an extensive review of medical records. Third, given the longitudinal nature with long follow‐up time is such that the quality of the endoscopic imaging has significantly changed during the study period, which could have impacted the rate of dysplasia detection in the early phase. On the other hand, this did not translate into a lack of effectiveness on disease‐specific mortality, which suggests that adherence to guidelines recommendation for biopsy and frequency of surveillance is paramount to ensure good outcome. There were also several strengths. First, this is one of the largest prospective cohorts published to date, with accurate endoscopic and pathologic correlation and longitudinal follow‐up. Secondly, all cases of dysplasia were reviewed by expert GI pathologists with research interest in BE, which ensures robustness of dysplasia diagnosis. Third, we performed an intention‐to‐treat analysis with interrogation of regional cancer registry for cancer diagnosis and disease‐specific mortality to reduce bias on loss in follow‐up.

In conclusion, this study indicates that endoscopic surveillance in expert centers is associated with a high detection rate of HGD and early cancer and low disease specific mortality and calls for further research to explore diagnostic quality indicators in BE surveillance.

## Author Contributions

Data collection: J.H., W.K.T., V.L., V.G., I.M.G., A.A.S., S.V., C.C., I.M., V.S. and M.dP.; Data analysis: J.H., W.K.T., V.L. and M.dP.; Database development: C.C.; Endoscopic procedures: J.H., W.K.T., I.M., V.S., M.dP.; Study conception: M.dP. and R.C.F.; Drafting manuscript: M.dP. and J.H.; All authors reviewed the final version of the manuscript and final approval of the version to be submitted and any revised version to be published.

## Conflicts of Interest

The authors declare no conflicts of interest.

## Supporting information

Table S1

## Data Availability

The data that support the findings of this study are available on request from the corresponding author. The data are not publicly available due to privacy or ethical restrictions.
