# Supplementary material for: Surveillance of Barrett's Esophagus Patients in an Expert Center is Associated With Low Disease‐Specific Mortality
Source: United European Gastroenterol J. 2025 Feb 13;13(2):220–8. doi: 10.1002/ueg2.12759 (PMC11975633; doi:10.1002/ueg2.12759)
Supplement: Supplementary file 1 — Table S1 [file UEG2-13-220-s001.docx]

**Supplementary Table 1. Summary of findings of longitudinal prospective studies in Barrett’s esophagus**

| Study | Country | Design | Setting | Patient number | Number of Progressors | Yearly rate |
| --- | --- | --- | --- | --- | --- | --- |
| Cambridge | UK | Prospective | Tertiary | 980 | 109 (11.2%) | 1.63% |
| Klaver et al | Netherlands | Prospective | Community | 985 | 65 (6.5%) | 0.78% |
| Kastelein et al. | Netherlands | Prospective | Mixed | 738 | 53 (7.1%) | 1.2% |
| Parasa et al | US (5) – Netherlands (1) | Prospective | Tertiary | 2697 | 154 (5.7%) | 0.95% |
